# Implicit Grammatical Gender Representation in Italian Children with Autism without Intellectual/Language Disorder

**DOI:** 10.3390/children10111737

**Published:** 2023-10-26

**Authors:** Caterina Artuso, Carmen Belacchi

**Affiliations:** 1 Department of Education, University of Genova, 16128 Genova, Italy; 2 Department of Communication, Humanities and International Studies, University of Urbino Carlo Bo, 61029 Urbino, Italy; carmen.belacchi@uniurb.it

**Keywords:** grammatical gender, children with autism, implicit language assessment

## Abstract

Grammatical language development in individuals with autism (without intellectual/language impairment) is mostly qualitatively comparable to language development in typically developing children of the same age. The majority of tasks used to study grammatical development require explicit performance (use of verbal language). Here, we administered an implicit categorization task (by biological sex) to understand which markers children use to implicitly infer grammatical gender representation in Italian (a gendered language where grammatical gender can be inferred via a determiner and/or word ending). Participants were asked to categorize photos of animals, relying on the names that differed in regard to the grammatical markers involved (i.e., lexical semantic, phonological, syntactic or phonological + syntactic). Children with autism displayed the same patterns observed in typically developing children: the lexical–semantic marker was categorized more accurately, followed in decreasing order by the phonological–syntactic marker and the phonological marker. The syntactic marker was the most difficult to categorize for both groups. In addition, children with autism showed an advantage in grammatical gender representation when using formal/grammatical markers than when using lexical/semantic markers. Such an implicit assessment allows for the investigation of more nuanced linguistic representations other than those expressed by traditional assessments.

## 1. Introduction

### 1.1. Autism and Language: An Overview

Autism is a neurodevelopmental condition characterized by disorders in communicative and social skills and restrictive/repetitive behaviors (according to [1]). The majority of research on language use in autism has focused on pragmatic difficulties [2,3], while less is known about the formal/structural aspects of language. Work on semantics, syntax and phonology is scarce and a lot is still to be understood about variations in grammatical competence profiles in autism, and the relationship between grammar competence and other cognitive abilities, as well [4]. 

### 1.2. Autism without Language Disorder

There is significant heterogeneity in the phenotype of individuals with autism, ranging from mild to more severe disorders. This range has been observed in the language skills of children with autism, as well: some show preserved language skills, while others develop little or no language skills [5]. Within language skills, pragmatic abilities have been found to be considerably poor, whereas grammatical abilities can vary widely. Thus, some children show average grammar [6], while others have notable difficulties with grammar. For instance, children with autism have been reported as experiencing a delay in producing their first words [7], first sentences [8], difficulties in producing grammatically correct morphemes for plurals and verb tenses [9], global syntactic disorder notwithstanding unimpaired lexical knowledge [10] and, overall, a smaller expressive vocabulary [11].

Moreover, it is worth mentioning the different trajectories of language development: in the first years of life, children with autism have been reported as having a “flatter language development” [12] than typically developing children; that is, language development proceeds more slowly in children with autism than in typically developing children of the same age. However, examination over a longer period of time shows that the trajectories can also be steeper. After an initial delay, there is accelerated growth. So, the existence of specific nuances in language development rather than gross measures points to a form of language development that proceeds in the same order and is qualitatively comparable to the course of language development in same-age non-autistic children [12].

These findings seem to be well accounted for by the arguments in [13]. Naigles [13] suggests that children’s acquisition of semantics (such as verb meanings and meaning-dependent structures) is more difficult than their discovery of the formal aspects of grammar (such as basic word order or identification and use of nominal morphology). Indeed, conceptual representations require the ability to share conventional representation via social interaction and the integration between social and physical stimuli to be efficiently processed, which is the specific domain where children with autism are particularly impaired (i.e., the semantic and pragmatic areas of linguistic development, e.g., [3]). Therefore, since the discovery and abstraction of grammatical forms usually occur prior to the complete establishment of their meanings, children with autism usually do not show as serious delays in grammatical development as they do for semantic and pragmatic development (see also [14]).

In their article entitled “Form is easy, meaning is hard”, ref. [14] summarizes the finding that the development of the formal/structural aspects of language (such as syntax and morphology) seems easier than the development of vocabulary/the lexical–semantic organization of knowledge. This could be related to the fact that learning formal/grammatical regularities is more in line with the characteristics of autism, such as attention to regularities and repetitiveness (e.g., [15,16]).

### 1.3. Autism and Language in Italian Children: Explicit vs. Implicit Assessment

Explicit language assessment measures require knowledge and use of language; for instance, we refer to traditional expressive and receptive language measures, such as word/non-word repetition, or general language production. Most of the tasks used to evaluate language development in autism (either via standardized batteries or nuanced ad hoc tests) require the explicit use of linguistic stimuli. For instance, linguistic development in Italian children was investigated via the production of pronouns/verb inflections [17] and receptive grammatical performance [18], both requesting the explicit use of language.

This assessment, that requires the use of language, may not be fully adequate to evaluate autism development, since it measures explicit language performance, one of the areas where children do show a communicative/linguistic disorder. Therefore, given the delayed social communication and interaction that characterize autism and, therefore, the clear impairment in this area, it would be of great interest to develop additional assessment procedures that evaluate language implicitly, or to assess the implicit representation that underpins explicit language mastery [19].

For this reason, we believe it would be of great usefulness to utilize tasks to investigate implicit language knowledge, considered a prerequisite for explicit language competence, in children with both typical and atypical development. Learning about regularities starts from implicit knowledge; afterwards, formal education boosts metalanguage knowledge and, subsequent, awareness of these patterns [19].

Related to the development of implicit knowledge, an area of investigation is the formal representation of the grammatical gender of nouns, a little-studied aspect of formal grammar development. This is a domain that is particularly salient for gendered languages, such as Italian, that is, a language where a noun’s grammatical gender is identified by a phonological marker (i.e., gender-marked ending) and/or a syntactic marker (i.e., gender-marked determiner), with such methods being used more and more given their possibility to detect more basic processes and mechanisms than traditional assessments.

### 1.4. Grammatical Gender Representation: An Overview

Grammatical gender symbolizes an arbitrary categorization system to distinguish nouns into two or more classes (i.e., female and male, such as in Italian, or female, male and neutral, such as in German) [20,21]. The same concept can be addressed via nouns of different genders in different languages. For instance, the same word may be feminine or masculine depending on the language (e.g., the word ‘the sun’ is feminine in German, ‘die sonne’, and masculine in Italian, ‘il sole’) [22].

Nevertheless, gender classification can be rather regular, being based on salient semantic properties of the referents (such as biological sex) and/or formal characteristics. Formal regularities correlated with the grammatical gender are frequently observed, although the criteria for such regularities vary according to different languages.

### 1.5. Grammatical Gender Representation in Italian

Italian, as with other Romance languages (e.g., French, Spanish), and unlike English, is characterized by grammatically marked nouns both for masculine and feminine (neuter is absent). Italian is a highly shallow language; indeed, it is characterized by stable and strong grapheme–phoneme correspondence. Therefore, Italian represents a key language for studying children’s representations of grammatical gender and their ability to process phonological and morpho-syntactic gender markers implicitly, as well.

Word suffixes assume a crucial role in determining the grammatical gender of Italian nouns. In Italian, the grammatical gender is expressed via the gender-marked word ending or the gender-marked determiner. Indeed, masculine nouns usually end with the vowel -o, whereas feminine nouns end with the vowel -a. Moreover, the masculine determiner “il/lo” (the) usually identifies a masculine noun (e.g., il tavolo (‘the table’)), whereas the feminine determiner “la” (the) usually identifies a feminine noun (e.g., la sedia (‘the chair’)) [23].

Children’s categorization abilities can then be modulated by implicit knowledge of grammatical gender, starting from pre-school age. At about 2;6 years of age, children master the notion of biological gender [24] and between 5 and 7 years recognize the invariance of gender identity [25]. However, as early as 12 months of age, Italian infants acquire and use the determiner gender features to improve comprehension and make predictions using a sentence’s words [26]. Grammatical gender effects have been observed in categorization tasks in Spanish [21], French [27], and Italian [28]; these effects appear earlier in transparent morphology languages (such as Italian) than in opaque morphologies (such as English or French). Overall, research indicates that children can implicitly use grammatical gender to categorize several types of stimuli, but findings concerning what formal cues they rely on are still unclear.

Specifically related to Italian, this categorization ability was studied by devising an implicit categorization task [28]. Sixty-four colored photos of animals were shown to children: half with feminine names and the other half with masculine names. They were then asked to name the animals represented in the photos; this was conducted to guarantee a preliminary control on their verbal production skills. Thus, this preliminary assessment is in line with the literature and represents an explicit language measure. Whether the child had correctly named the animal or not, the experimenter next stated the animal noun without any determiner (e.g., ‘The name of this animal is lion’—“leone”). All the children were then provided with the correct noun corresponding to each animal photo. Therefore, the children could categorize the animal word only via the grammatical features of the word (i.e., phonological, syntactic, or phonological–syntactic). No other linguistic information was provided.

Afterwards, the children were presented with a forced choice task. If they thought an animal was a male, they were invited to place the photo in a blue box; on the contrary, if they thought an animal was a female, they were invited to place the photo in a pink box. Importantly, participants were not requested to identify the grammatical gender of the animal in the photos, but to categorize them as male or female according to the perceived biological sex, inferable only by their name (but there were three cases of lexical–semantic stimulus words, where sexual differences are specific and identify males and females, that is: rooster/hen, bull/cow, lion/lioness). All the other photos and, in particular, those with epicenes nouns, did not provide any cue attributable to the sex of the animal depicted. Thus, to perform the task, the children could only rely on grammatical gender indices for the name of the animals (word ending and/or determiner retrieval).

Three- to five-year-old preschool children recognized word gender [28]; more specifically, they better categorized lexical–semantic stimulus words (i.e., nouns whose gender corresponded to the sex of the animal: e.g., leone vs. leonessa, lion, lioness), followed by phonological–syntactic stimulus words (i.e., nouns with both a gender-marked ending and a determiner: e.g., il pinguino vs. la giraffa, penguin, giraffe), and phonological stimulus words (i.e., nouns with a gender-marked ending, without an informative gender determiner, for instance (l’) agnello or (l’) aquila, lamb, eagle). For these children, the most difficult type of indices were the syntactic ones (i.e., nouns with a gender-opaque ending (e) and with a gender-marked determiner only: il rinoceronte vs. la tigre, rhinoceros, tiger). See Table 1.

Further support for the findings by [28] was reported by [29], who standardized in Italian preschoolers the categorization task and [30]. It is worth noticing that the task previously described [28] assessed linguistic representations implicitly, that is not asking for any language performance, thus selecting and retrieving the correct determiner for each name (except the preliminary naming task, which represented a control on language production). Indeed, the task was aimed at testing the spontaneous linguistic representation of grammatical gender owned by the child. Thus, children must categorize animal photos by their biological sex (e.g., ‘pantera’—panther—as female); afterwards, their responses were treated as an index of the implicit mental representation of grammatical gender. Indeed, grammatical gender representation can be accomplished only using implicit knowledge of the animal names (e.g., ‘pantera’ is a feminine epicene as the gender-marked ending is a feminine one, -a, and requires the feminine determiner la).

Another work investigated the representation of grammatical gender in children with autism. The acquisition of grammatical gender in Arabic was studied [31], using an explicit task where children were requested to conclude brief sentences composed of a noun and an adjective agreeing with the former in regard to its gender (i.e., a target agreement task). Children could complete the task via the help of illustrative pictures. This task was conducted before and after a 3-week training (on gender agreement) on the procedure, finding a beneficial effect of pictures as an effective procedure in teaching children with autism.

Apart from this study, which was conducted in the Semitic language and used an explicit task, we have no knowledge of other tasks that implicitly investigate the role of grammatical markers (i.e., semantic and/or phonological–syntactic) in the construction of grammatical gender representation in individuals with autism.

### 1.6. The Current Study

As previously described, the first study was conducted with children by [28], who devised an implicit categorization task for studying implicit grammatical gender representation. In addition, two further studies using the same task were conducted. Ref. [29] standardized the task for Italian preschoolers, defining norms for children from 3 to 5 years of age. In a second study [30], it was investigated the implicit grammatical gender representation in children with developmental language disorder, who are usually evaluated via explicit language assessment (e.g., word/non-word repetition tasks). Interestingly, the results showed that while typically developing children were better at using phonological markers of animal names to categorize the photos, children with a developmental language disorder used phonological gender markers less accurately.

The aim of the current study was, therefore, to investigate how children with autism (without intellectual/language disorder) performed an implicit categorization task [28], to observe which patterns they displayed in grammatical gender representation. Children with autism were tested to better understand the nature of language acquisition in regard to both the semantic and morpho/syntactic aspects and to better identify the analogies and specificities of children with autism compared to typically developing children. Disorders in regard to the semantic/pragmatic aspects are documented, less is known about the morpho-syntactic aspects.

Therefore, the representation of grammatical gender is especially crucial and interesting in gendered languages, such as Italian, where it is possible to clearly distinguish the semantic from the phonological and syntactic aspects, which could contribute to the clarification of language development in this clinical population.

In line with previous findings [28,29], we predicted that the lexical–semantic marker would be categorized more accurately, followed in decreasing order by the combination of the phonological–syntactic marker and the phonological marker in the typically developing group (TD). The syntactic marker should be the most difficult to categorize for both groups. Indeed, as the literature points out, grammar proficiency in children with autism follows the same patterns found in same-age peers [4,13,14]. It is worth noting that most of the tasks used to study grammatical development require explicit performance (i.e., require the use of verbal language either in production or repetition or comprehension). Here, on the contrary, we used an implicit task (i.e., the categorization of animals via their nouns without a determiner, already standardized and used in typical development and in developmental language disorders) to assess which lexical–semantic and formal markers individuals use to represent grammatical gender.

In line with the literature (see [4,13,14]), we expected the same pattern of answers in children with autism (without intellectual/language disorder) and in the TD group, that is, better biological sex categorization for lexical–semantic stimulus words, followed by phonological–syntactic and phonological ones. The syntactic stimulus words should be the most difficult to categorize [28,30]. In addition, we expected children with autism to underperform in the categorization of lexical–semantic stimulus words. These represent a formally heterogeneous category of stimulus words that can be learnt via sharing conventional semantic representations and not via learning the formal regularities of a given stimulus word. Thus, because children with autism usually show difficulties in sharing conventional meanings [14], we predicted lower scores than in the TD group.

A further objective, in line with the literature suggesting that the acquisition of concepts/meanings is more difficult than the identification of formal aspects of grammar [14], was to investigate whether such an effect is observable in an implicit task, as well. Specifically, we compared a semantic/global strategy (i.e., the categorization of pictures using the associated lexical/semantic knowledge) to a formal/analytic strategy (i.e., the categorization of pictures using the associated formal knowledge). Since children with autism are more used to systematization and pay particular attention to regularities (e.g., [15,16]), then they should have an advantage in categorizing using formal markers than in categorizing using lexical/semantic markers.

## 2. Materials and Methods

### 2.1. Participants

Fifty-six Italian Caucasian participants shared between two groups (28 each) were tested. The mean age was of 8;2 years (*SD* = 2;9), with a range of 3;1–13;1 years. In each group, there were 23 males. All the children were monolingual, and none had a hearing impairment. The children with autism (without intellectual/language disorder) were recruited from three different clinical centers located in central Italy (i.e., in Fano, Marche; in Perugia, Umbria; in Atri, Abruzzo). These are clinical centers with high specialization in autism and, more specifically, are centers accredited to diagnose and certify autism. Following our requests, the neuropsychiatrists in charge selected only the children without an intellectual/language disorder.

These children, indeed, showed average scores for cognitive and linguistic development. Specifically, for receptive language the Peabody Picture Vocabulary Test–Revised was administered (mean score of 83, *SD* 8.9, range 78–101). This showed average scores for language comprehension. In addition, we had an explicit language production measure embedded in the categorization task that showed no differences between the groups, that is regarding the preliminary control naming task (see Section 3). This ensured the two groups did not differ in their verbal naming abilities.

Moreover, we obtained some cognitive measures of general intelligence that differed by age group. Specifically, for 9 participants aged 3;0 to 5;9 years, we obtained the global IQ derived from the WPPSI administration (mean score 120, *SD* 19.09, range 107–134); for the other 23 participants aged 6;1 to 13;1 years, we obtained the global IQ derived from the WISC IV administration (mean score 95, *SD* 12.35, range 80–107). In addition, all our participants were administered the Leiter test as a measure of non-verbal/fluid intelligence (mean score 94.83, *SD* 8.32, range 79–102), shown to be sensitive in testing specific weaknesses in high-functioning autistic children [32]. Overall, our group of children with autism showed average cognitive/linguistic profiles.

We compared the children with autism to a group of Italian children recruited from public schools who had not been diagnosed with any cognitive or language disorder and were matched in terms of age, gender and school level (no significant differences were observed, *p* > 0.05). In both groups, given that the age range was broad, we created two subgroups of younger and older children, to have a complete picture of possible age-related changes. The subgroups were similar in regard to the number of children; therefore, we had 13 younger (aged from 3;1 to 7;6 years) and 15 older children (aged from 8;1 to 13;1 years). Specific data on the socioeconomic status were not recorded.

The study was conducted according to the ethical guidelines by the Italian Psychological Association. Ethical approval was obtained from the University of Urbino (approval number: RCE-1/05).

### 2.2. Materials

The two groups of participants were administered the categorization task, previously described [28]. For Italian norms and the full list of original animal photos see [29]. The task showed good psychometric properties, with a split-half reliability of 0.80.

Sixty-four color photos of animals were presented to the children, named by the experimenter without the determiner. They had to categorize the animals as male or female in agreement with their perceived biological sex. The animal nouns presented in the photos and pronounced by the experimenter differed in regard to the linguistic markers of grammatical gender. The stimulus words were distinguished into four groups: lexical–semantic, phonological–syntactic, phonological and syntactic.

The groups of stimulus words were matched neither for word frequency nor familiarity, because nouns from the lexical–semantic group were more frequent and more familiar than the nouns belonging to the other three categories, with all *p* < 0.001 (i.e., the epicenes; cfr. norms from the elementary lexicon of Italian [33]). However, it is worth noting that the frequency did not significantly differ between the three other groups of stimulus words (*p* > 1). Here is the mean frequency rank for each group of stimulus words, taken from [33], considering that the higher the rank, the less frequent the word is, and the lower the rank, the more frequent the word is: lexical–semantic (rank: 823), phonological–syntactic (rank: 2862), phonological (rank: 3154) and syntactic (rank: 2845).

### 2.3. Procedure and Scoring

All the children were tested individually in a room at the clinical center (for children with autism) or at school (for the TD group). For a full description of the procedure, see the introduction. Sixty-four color photos of animals were shown to children, presented on a uniform white background, in a random order: half of the animals had feminine names and the other half had masculine names (as exemplified in Table 1). The order in the presentation of the photos was not blocked by type but interspersed; also, the presentation order varied for each child.

At the beginning of the experimental session, three practice photos were shown to familiarize the children with the procedure. The experimenter ensured the child knew the difference between the male and female gender, and that s/he was able to grasp the color coding on the gender difference and apply it to animals.

The response was “correct” only if in agreement with the grammatical gender of the animal noun. One point was assigned for each answer that was correct (i.e., congruent with the grammatical gender of the animal noun); zero points were assigned for each incorrect response. For the overall task, the possible range of scores was 0–64; and 0–16 for the lexical–semantic and syntactic markers, 0–17 for the phonological–syntactic markers and 0–15 for the phonological markers (see Appendix A for the full list of stimulus words). Given that the four categories were not perfectly comparable in regard to the number of stimulus words, we calculated the mean proportional scores to compare them in the analyses. Latencies of the responses were not considered, and “I don’t know” responses were not admitted in the task.

## 3. Results

### 3.1. Preliminary Control Task: Naming Task

As a preliminary control on explicit language production, we tested the two groups of children on naming task performance. In fact, as previously described, children were asked to name the photo of the animal they were presented with. This was a control on the verbal competence of the two groups and was conducted to obtain a measure of explicit lexical production and ensure the two groups were comparable. Descriptive statistics are reported in Table 2.

An analysis was run with the group (TD, children with autism) and age (younger, older) as the between-participants factor and the grammatical gender marker (lexical–semantic, phonological–syntactic, phonological, syntactic) as the within-participants factor, on the mean proportional accuracy of the correctly named animal names. The main effect of age reached significance, F (1, 52) = 19.94, *p* < 0.001, η²_p_ = 0.28, showing greater accuracy in older children (M = 0.51, SD = 0.02) than in younger children (M = 0.40, SD = 0.02). Age did not interact significantly with any other factor.

No significant differences between the two groups were found, F (1, 52) = 0.36, *p* = 0.55. Indeed, both groups named the animals with the same accuracy. The type of grammatical gender marker wielded a significant effect, F (3, 50) = 36.26, *p* < 0.001, η²_p_ = 0.69. Pairwise comparisons of the overall mean showed that both groups of children were better at naming based both on the lexical–semantic marker and the phonological–syntactic marker (these did not differ, *p* = 0.74; lexical–semantic M = 0.56, SD = 0.01; phonological–syntactic M = 0.55, SD = 0.02). Both these markers produced better verbal naming than the phonological (M = 0.33, SD = 0.02) and syntactic markers (M = 0.38, SD = 0.01) (all *p* < 0.001). The phonological and syntactic markers did not differ, *p* = 0.065. Moreover, the interaction between the grammatical gender marker and the group was not significant, F < 1.

### 3.2. Experimental Task: Categorization Task

An analysis was run with the group (TD, children with autism) and age (younger, older) as the between-participants factor and the grammatical gender marker (lexical–semantic, phonological–syntactic, phonological, syntactic) as the within-participants factor, on the mean proportional accuracy of the correctly categorized animal names. No significant differences between the two groups were found, F (1, 52) = 1.62, *p* = 0.21. Indeed, both groups categorized the same number of animal names with similar accuracy (M = 0.75, SD = 0.02 for the TD group, and M = 0.78 SD = 0.02 for the children with autism). Descriptive statistics are reported in Table 3.

The main effect of age reached significance, F (1, 52) = 14.85, *p* < 0.001, η²_p_ = 0.22, showing greater accuracy in older children (M = 0.82, SD = 0.02) than in younger children (M = 0.72, SD = 0.02). Age did not interact significantly with any other factor.

**Figure 1:**
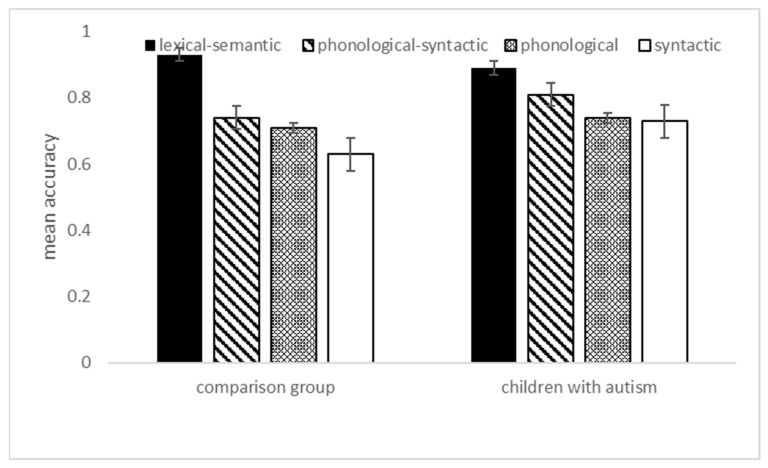
Categorization task: mean proportional accuracy by grammatical gender marker. Bars represent SEM.

The type of grammatical gender marker wielded a significant effect, F (3, 50) = 29.16, *p* < 0.001, η²_p_ = 0.36. The interaction between the group and the grammatical gender marker was significant, as well, F (3, 50) = 3.46, *p* = 0.018, η²_p_ = 0.06, and is represented in Figure 1.

Paired-sample comparisons were conducted within each group, to obtain a detailed pattern on linguistic marker use. In the TD group, lexical–semantic markers led to greater accuracy than the phonological–syntactic ones, t(27) = 12.37, *p* < 0.001; moreover, the lexical–semantic markers produced better scores than both the phonological markers, t(27) = 6.22, *p* < 0.001, and the syntactic markers t(27) = 8.28, *p* < 0.001. Likewise, the phonological–syntactic markers led to greater accuracy than the syntactic markers, t(27) = 2.32, *p* = 0.028, but no difference in accuracy was found with the phonological markers (*p* = 0.60). No significant differences were found between the phonological and syntactic markers (*p* = 0.18).

In children with autism, the lexical–semantic markers were associated with greater accuracy than both the phonological–syntactic markers, t(27) = 2.78, *p* = 0.010, the phonological markers, t(27) = 3.30, *p* = 0.003, and the syntactic ones, t(27) = 4.38, *p* < 0.001. The phonological–syntactic markers were associated with greater accuracy than the syntactic ones, t(27) = 2.58, *p* = 0.016, but no difference in accuracy was found with the phonological markers (*p* = 0.60). No significant differences were found between the phonological and syntactic markers (*p* = 0.67).

Independent sample comparisons showed no differences between the two groups in regard to all the markers, as it can be observed from Figure 1, as well (all Fs < 1).

### 3.3. Use of Strategies in the Categorization Task

A further analysis was run with the group (TD, children with autism) and age (younger, older) as the between-participants factor and the strategy (semantic, formal) as the within-participants factor, on the mean proportional accuracy of the correctly categorized animal photos in accordance with their name. The semantic strategy was represented by the mean proportional accuracy obtained from the lexical–semantic names (i.e., names whose gender corresponds to the biological sex of the animal). The formal strategy was represented by the mean proportional accuracy obtained from the other three markers (which have been collapsed into a single measure), where it is crucial to rely on the formal aspects of grammar, that is phonological–syntactic or both.

No significant differences were found between the two groups, F (1, 52) = 0.006, *p* = 0.98. The main effect of age reached significance, F (1, 52) = 10.38, *p* = 0.002, η²_p_ = 0.17, showing greater accuracy in older children (M = 0.86, SD = 0.02) than in younger children (M = 0.77, SD = 0.02). Age did not interact significantly with any other factor.

The strategy interacted with the group, F (1, 52) = 17.65, *p* < 0.001, η²_p_ = 0.25. Paired-sample comparisons were then conducted within each group to obtain a more detailed pattern on strategy use. In the TD group, the semantic strategy led to greater accuracy than the formal one, t (27) = 14.91, *p* < 0.001; similarly, in children with autism, the semantic strategy produced greater accuracy than the formal one, t (27) = 4.50, *p* < 0.001.

**Figure 2:**
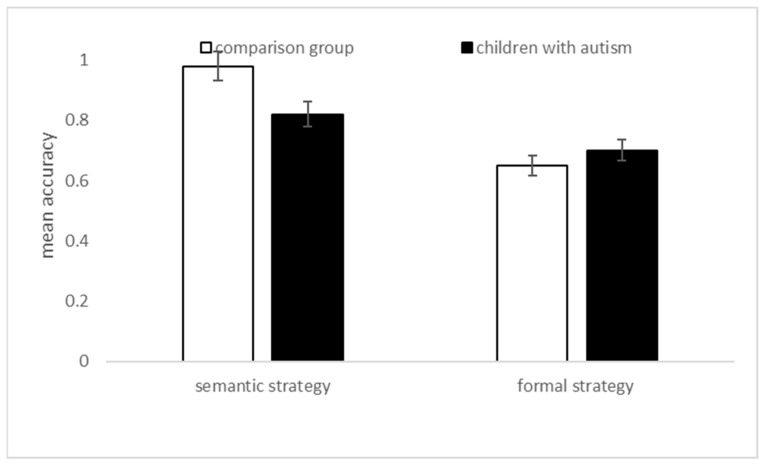
Categorization task: mean proportional accuracy by strategy use. Bars represent SEM.

Notwithstanding, and more originally, the independent sample t-tests showed no differences in the use of semantic strategy between the two groups (t (54) = 1.68, *p* = 0.10; Cohen’s d = 0.44, with a small effect); on the contrary, the formal strategy was mainly used by children with autism than the children from the TD group (t (54) = 2.13, *p* = 0.037; Cohen’s d = 0.58, with a small-to-medium effect), as represented in Figure 2.

## 4. Discussion

Social interaction and communication/pragmatics disorders mainly characterize autism [2,3,12]. The current perspective on language development argues that language is mostly qualitatively comparable to the course of language development in non-autistic children of the same age (see [12]). Indeed, language use by autistic children can be delayed, both in regard to the expressive and the receptive formal grammar components, when compared to TD [4,7,10]. Autistic children’s acquisition of semantics is more difficult than their discovery of the formal/structural aspects of grammar and syntax [13,14].

In the current study, a categorization task was administered to children with autism (without intellectual/language disorder) to evaluate their implicit grammatical gender representation [28,29,30]. The tasks traditionally used to assess linguistic/communication skills in autism, as well as in children with typical development, are explicit, that is, they involve verbal skills and require comprehension and the production of verbal tenses, sentences or similar [18]. In addition, tasks used to evaluate grammatical gender representation are scarce and use different measures, such as sentence completion or gender agreement tasks [31]. However, in our view, these explicit linguistic tasks might be perceived as difficult and unenjoyable; therefore, children may be unlikely to feel motivated to engage with these tasks. For this reason, here, we used a task that measures implicit linguistic competence, while bypassing explicit linguistic knowledge and, thus, providing a view on the development of underpinning grammatical representations.

The task administered [28] included four different grammatical gender markers: lexical–semantic, phonological–syntactic, phonological and syntactic (exemplified in Table 1). Ref. [28] found that typically developing children were able to recognize a word’s gender and they easily used lexical–semantic markers, followed by phonological–syntactic markers and phonological markers; syntactic markers were found to be the most difficult. The results observed in the TD group confirmed previous findings obtained by [28,29,30].

The aim of the naming task was to obtain a measure of the explicit verbal competence and make sure the two groups were comparable; this was demonstrated by the results (mean naming performance was 0.46 for TD and 0.45 for children with autism, respectively; see also Table 2). The fact that the naming performance was low, we believe, constitutes a strength of our task; indeed, although the children were not able to correctly name all the animal photos, they were able to categorize them by gender. Thus, they were able to implicitly use formal gender markers to categorize words, also when they did not know the exact meaning of the word. We believe it would also be possible to obtain the same results when using ad hoc created words; in this vein, the importance of implicit language learning via statistical regularities on verbal stimuli in typical preschool children was shown in [34]. Given the specific sensitivity of children with autism to statistical regularities in general [15,16], we believe this could be a future study to design.

As far as the semantic categorization is concerned, interestingly and originally, in children with autism (without intellectual/language disorder), we found the same patterns previously observed in the TD group, with the lexical–semantic markers more frequently used than the others, followed by the combination of the phonological–syntactic markers, phonological markers and, lastly, the syntactic markers (the most difficult). See Table 3 for full descriptive statistics (the mean categorization performance was 0.75 for TD and 0.78 for children with autism, respectively). These findings, we believe, are in line with the literature that claims that for an autistic child formal/structural language development is qualitatively comparable to the course of language development in non-autistic children of the same age [12]. Thus, as represented in Figure 1, the two groups showed overlapping patterns of grammatical gender representation development, confirming previous studies (e.g., [4]).

It Is worth noticing the absence of differences between the two groups (TD and children with autism) in the lexical–semantic stimulus words categorization. We predicted that children with autism would underperform in the categorization of lexical–semantic stimulus words, as these represent a category of stimuli that can be learnt via sharing conventional semantic representations, a field where children with autism usually show fragilities (e.g., [13,14]). This unexpected finding is coherent with a recent distinction that has been observed in children with autism, between semantic and pragmatic communication, that until now were associated [35]. Indeed, these preliminary findings could support a pragmatic communication deficit, but not a semantic one.

It should also be noted, the different frequency of the four groups of stimulus words with the lexical–semantic being the most frequent than any other stimulus type (see Section 2). In general, the lexical–semantic markers facilitate categorization by both groups (compared to the other three categories; see Figure 1).

The syntactic marker is more difficult for both groups; in addition, it is worth noting that it is equally impaired when compared to the phonological marker. Moreover, according to the developmental literature, the determiner is produced at around 2 years of age, but it is acquired before [26]. Therefore, it is likely that the difficulties are not only related to the determiner, but also to the opaque (irregular) ending. It is also worth noting that the greater difficulty lies in the fact that, whereas the shallow word ending is already available in the stimulus, the determiner needs to be inferred; therefore, additional cognitive resources are required, such as linguistic memory.

We believe it is also worth mentioning the discriminant validity of this task, as well as the theoretical assumptions. The gender categorization task was administered to children with a developmental language disorder [30], finding a peculiar difficulty in using phonological markers of gender. On the contrary, in the current study, we did not find impairment in a specific formal grammatical gender marker, either phonological (i.e., gender-marked ending) or syntactic (i.e., gender-marked determiner). When assessed via a more nuanced task (rather than a global assessment), children with autism are not impaired in the language component of grammatical gender categorization and show a level of development comparable to same-age comparison groups. This result (obtained via an implicit assessment task) is also in line with current research that demonstrates that in developmental language disorders, standardized tests are not necessarily sensitive to the different types of grammatical impairments typically observed [36]; thus, this could also be the case for children with autism, where exploring early implicit markers of language development might be useful [37].

Our second objective was to investigate which strategies individuals with autism use in an implicit task comparing a semantic/global strategy to a formal/analytic one. In line with the literature suggesting that these children’s acquisition of concepts/meanings is more difficult than their discovery of the formal aspects of grammar [13,14], we found that they are more sensitive to a formal strategy, which is useful to systematize formal components of language (such as determiners or endings), rather than the semantic aspects, such as those implied by lexical–semantic stimulus word representation (though the effect is small-to-medium, see Figure 2 and Section 3). This is in line with recent literature developments on how individuals with autism organize experiences (i.e., more local than global [38]), and via more systematic approaches than empathic ones [15,16]. In turn, this may contribute to accounting for specific pragmatic impairments in autism.

The tasks we described in the introduction refer to explicit language production and are, therefore, quite different from this current one, requesting an implicit evaluation of the names’ grammatical gender. So, it is not simple to compare the current findings to the existing literature. However, we can notice that a few studies showed a delay in producing their first words [7], first sentences [8], difficulties in producing grammatically correct morphemes for plurals and verb tenses [9] and global syntactic disorder. On the contrary, here, we showed an advantage in the use of the formal aspects of grammar in the scope of categorization (see Figure 2). Importantly, a direct comparison with the literature is not possible given the relevant tasks’ differences and objectives.

A few major limitations should be taken into account. First, our sample size was small; this should be taken into account and some caution is suggested when drawing conclusions. In addition, it would be useful to have more details about the sample recruitment and individual’s features to characterize more accurately individuals’ characteristics that may impact language development. In addition, as noticed (see Section 2), the lexical–semantic stimulus words are more frequent, compared to the other three categories; however, we believe this does not represent a limitation because we were interested in the between-group comparison (individuals with autism vs. typically developing individuals). Moreover, additional linguistic measures should be used to better characterize the groups.

As a future development, we plan to carry out a twofold assessment of language, both explicitly (e.g., word repetition or production) and implicitly (e.g., implicit assessment like the one we used here), using the same sample of individuals to have a direct comparison of the development of these two features of language: the former explicit, in line with traditional assessments, the latter implicit, an index of the mental representation behind language development.

In addition, to add further support to the dissociation formal grammar vs. semantic/conceptual knowledge, it might be of interest to manipulate these two levels within different domains. For instance, considering the poor executive functioning of individuals with ASD, they could be administered working memory tasks where the phonological/orthographical level is manipulated (formal grammar [39,40]), as well as where the semantic association is manipulated (semantic knowledge).

In conclusion, we showed a comparable trend in the pattern for implicit grammatical gender representation between children with autism (without intellectual/language disorder) and the TD group. Moreover, we demonstrated that such an implicit language assessment modality allows the investigation of more nuanced linguistic representation other than those expressed by traditional assessments. On the one hand, we confirmed a delay in semantic processing; on the other hand, we showed better sensitivity in the formal aspects of language processing. In turn, this could be helpful to unveil key strengths through different linguistic impairments observable in clinical groups, such as genetic syndromes. Also, since the severity of autism was found to impact children’s language processing (especially syntactically complex material, see also [41]), it would be valuable to precisely assess and intervene early in regard to linguistic impairments and/or promote different aspects of linguistic ability that peculiarly characterize individuals with different cognitive profiles.

## Figures and Tables

**Table 1:** Italian stimulus word examples (with English translation).

Grammatical Gender Marker	Stimulus Words
**Lexical–semantic**. Nouns with the gender corresponding to the animal’s biological sex	toro (bull, *m*) mucca (cow, *f*)
**Phonological–syntactic**. Epicene showing both gender-marked ending (a/o) and gender-marked determiner (il/la)	(la) mosc*a* (fly, *f*) (il) delfin*o* (dolphin, *m*)
**Phonological**. Epicene showing gender-marked ending (o/a) and the determiner is not informative (l’)	(l’) anatr*a* (duck, *f*) (l’) agnell*o* (lamb, *m*)
**Syntactic**. Epicene showing gender-opaque ending (e) and gender-marked determiner (il/la)	(il) serpent*e* (snake, *m*) (la) volp*e* (fox, *f*)

Note: *f* is for feminine; *m* is for masculine.

**Table 2:** Naming task: descriptive statistics (mean and SD in parentheses) for the four grammatical gender markers distinguished by group (TD, children with autism) and age (younger, older).

Group	TD	TD	Children with Autism	Children with Autism
	Younger	Older	Younger	Older
Lexical–semantic	0.49 (0.12)	0.68 (0.13)	0.50 (0.08)	0.55 (0.08)
Phonological–syntactic	0.46 (0.12)	0.57 (0.14)	0.49 (0.21)	0.61 (0.13)
Phonological	0.21 (0.11)	0.50 (0.23)	0.22 (0.20)	0.48 (0.14)
Syntactic	0.34 (0.10)	0.42 (0.10)	0.34 (0.15)	0.39 (0.09)

**Table 3:** Categorization task: descriptive statistics (mean and SD in parentheses) for the four grammatical gender markers distinguished by group (TD, children with autism) and age (younger, older).

Group	TD	TD	Children with Autism	Children with Autism
	Younger	Older	Younger	Older
Lexical–semantic	0.93 (0.07)	0.94 (0.04)	0.82 (0.22)	0.93 (0.10)
Phonological–syntactic	0.77 (0.13)	0.70 (0.03)	0.72 (0.24)	0.90 (0.13)
Phonological	0.64 (0.13)	0.78 (0.21)	0.66 (0.18)	0.82 (0.15)
Syntactic	0.56 (0.14)	0.70 (0.20)	0.65 (0.13)	0.80 (0.18)

## Data Availability

Data are available on request to the first/corresponding author.

## Appendix A

**Table A1:** Italian grammatical gender representation task.

Stimulus Word	Category	Grammatical Gender (In Italian)	Proportion of Correct Responses
Lupo (Wolf)	Lexical–semantic	Masculine	0.84
Cavallo (Horse)	Lexical–semantic	Masculine	0.84
Gatto (Cat)	Lexical–semantic	Masculine	0.74
Cane (Dog)	Lexical–semantic	Masculine	0.84
Toro (Bull)	Lexical–semantic	Masculine	0.79
Gallo (Rooster)	Lexical–semantic	Masculine	0.74
Leone (Lion)	Lexical–semantic	Masculine	0.84
Elefante (Bull elephant)	Lexical–semantic	Masculine	0.69
Lupa (She-wolf)	Lexical–semantic	Feminine	0.84
Cavalla (Mare)	Lexical–semantic	Feminine	0.74
Gatta (She-cat)	Lexical–semantic	Feminine	0.74
Cagna (Bitch)	Lexical–semantic	Feminine	0.53
Mucca (Cow)	Lexical–semantic	Feminine	0.58
Gallina (Hen)	Lexical–semantic	Feminine	0.58
Leonessa (Lioness)	Lexical–semantic	Feminine	0.69
Elefantessa (Cow elephant)	Lexical–semantic	Feminine	0.63
Falco (Hawk)	Phonological–syntactic	Masculine	0.69
Pappagallo (Parrot)	Phonological–syntactic	Masculine	0.83
Granchio (Crab)	Phonological–syntactic	Masculine	0.84
Delfino (Dolphin)	Phonological–syntactic	Masculine	0.79
Struzzo (Ostrich)	Phonological–syntactic	Masculine	0.74
Pinguino (Penguin)	Phonological–syntactic	Masculine	0.84
Cammello (Camel)	Phonological–syntactic	Masculine	0.84
Leopardo (Leopard)	Phonological–syntactic	Masculine	0.63
Foca (Seal)	Phonological–syntactic	Feminine	0.58
Pecora (Sheep)	Phonological–syntactic	Feminine	0.63
Pantera (Panther)	Phonological–syntactic	Feminine	0.63
Zebra (Zebra)	Phonological–syntactic	Feminine	0.58
Farfalla (Butterfly)	Phonological–syntactic	Feminine	0.79
Tartaruga (Tortoise)	Phonological–syntactic	Feminine	0.48
Giraffa (Giraffe)	Phonological–syntactic	Feminine	0.63
Mosca (Fly)	Phonological–syntactic	Feminine	0.69
Iena (Hyena)	Phonological–syntactic	Feminine	0.58
Procione (Raccoon)	Syntactic	Masculine	0.84
Piccione (Pigeon)	Syntactic	Masculine	0.90
Scorpione (Scorpion)	Syntactic	Masculine	0.74
Rinoceronte (Rhinoceros)	Syntactic	Masculine	0.84
Serpente (Snake)	Syntactic	Masculine	0.90
Scimpanzè (Chimpanzee)	Syntactic	Masculine	0.84
Camaleonte (Chameleon)	Syntactic	Masculine	0.84
Calabrone (Hornet)	Syntactic	Masculine	0.69
Rondine (Swallow)	Syntactic	Feminine	0.32
Pulce (Flea)	Syntactic	Feminine	0.37
Lepre (Hare)	Syntactic	Feminine	0.63
Pernice (Partridge)	Syntactic	Feminine	0.27
Mantide (Mantis)	Syntactic	Feminine	0.53
Volpe (Fox)	Syntactic	Feminine	0.48
Tigre (Tiger)	Syntactic	Feminine	0.63
Lince (Lynx)	Syntactic	Feminine	0.48
Agnello (Lamb)	Phonological	Masculine	0.58
Usignolo (Nightingale)	Phonological	Masculine	0.74
Allocco (Tawny owl)	Phonological	Masculine	0.79
Ornitorinco (Ornithorhynchus)	Phonological	Masculine	0.79
Orango (Orangutan)	Phonological	Masculine	0.74
Ippopotamo (Hippopotamus)	Phonological	Masculine	0.84
Armadillo (Armadillo)	Phonological	Masculine	0.79
Ermellino (Ermine)	Phonological	Masculine	0.69
Aquila (Eagle)	Phonological	Feminine	0.53
Otaria (Sea lion)	Phonological	Feminine	0.58
Oca (Goose)	Phonological	Feminine	0.74
Anatra (Duck)	Phonological	Feminine	0.63
Upupa (Hoopoe)	Phonological	Feminine	0.64
Aragosta (Lobster)	Phonological	Feminine	0.63
Iguana (Iguana)	Phonological	Feminine	0.53
